# Blood pressure and microvascular free flap perfusion in head and neck reconstruction– a retrospective analysis

**DOI:** 10.1007/s10006-025-01378-8

**Published:** 2025-04-21

**Authors:** Mark Ooms, Philipp Winnand, Marius Heitzer, Nils Vohl, Marie Sophie Katz, Johannes Bickenbach, Frank Hölzle, Ali Modabber

**Affiliations:** 1 Department of Oral and Maxillofacial Surgery, University Hospital RWTH Aachen, Pauwelsstraße 30, 52074 Aachen, Germany; 2 Department of Intensive Care Medicine, Uniklinik RWTH Aachen, Pauwelsstraße 30, 52074 Aachen, Germany

**Keywords:** Microvascular head and neck reconstruction, Free flap, Blood pressure, Blood flow, Hemoglobin concentration, Hemoglobin oxygen saturation

## Abstract

**Purpose:**

The influence of blood pressure on microvascular free flap perfusion is not fully understood and remains an ongoing topic of debate, as flap perfusion is both a prerequisite for flap viability and a parameter frequently used for postoperative flap monitoring. The aim of this study was to investigate the influence of blood pressure on microvascular free flap perfusion.

**Methods:**

Perfusion measurement data obtained with the Oxygen-2-see (O2C) analysis system intraoperatively and postoperatively in 244 patients who underwent microvascular reconstruction of the head and neck region with a fasciocutaneous free flap (FFF) or perforator free flap (PFF) between 2011 and 2020 were analyzed retrospectively. Blood pressure values (i.e., systolic (SBP), diastolic (DBP), and mean arterial blood pressure (MBP)) and perfusion parameters (i.e., flap blood flow, hemoglobin concentration, and hemoglobin oxygen saturation) were evaluated to reveal potential associations.

**Results:**

Postoperative flap blood flow was associated with SBP, DBP, and MBP in PFFs (*r* = 0.245, *p* = 0.006; *r* = 0.239, *p* = 0.008; *r* = 0.295, *p* < 0.001, respectively). These associations also persisted in multivariable analysis (*p* = 0.036; *p* = 0.024; *p* = 0.004, respectively). Postoperative hemoglobin oxygen saturation was associated with SBP and MBP in PFFs (*r* = 0.253, *p* = 0.005; *r* = 0.189, *p* = 0.036, respectively). The association with SBP also persisted in multivariable analysis (*p* = 0.005).

**Conclusion:**

Microvascular free flap perfusion in PFFs, specifically postoperative flap blood flow and hemoglobin oxygen saturation, is influenced by blood pressure. This suggests that blood pressure might be an adjustable variable for the control of flap perfusion and should be considered as a confounding variable for flap monitoring based on flap perfusion in PFFs.

## Introduction

Free tissue transfer with microvascular free flaps is routinely performed for the reconstruction of complex defects in the head and neck region, offering excellent outcomes [1–3]. Despite high overall success rates, flap failures still occur, and the underlying risk factors remain a current topic of discussion [1, 3–5].

Blood pressure has attracted attention as a potential risk factor, as low blood pressure regularly occurs intra- and postoperatively in microvascular reconstructive procedures, unintentionally due to the side effects of anesthetics and high levels of blood and fluid loss but also intentionally to reduce blood loss and improve the visualization of the surgical field [4, 6–12]. Meanwhile, blood pressure is thought to be a major determinant of flap perfusion, and because decreased flap perfusion has been linked to flap failure, low blood pressure could impair flap perfusion and contribute to flap failure [2, 6, 7, 11, 13–16].

However, it is still unclear whether and how blood pressure influences flap perfusion. One study affirmed the influence of blood pressure on flap perfusion based on tissue oxygen partial pressure, while another did not [17, 18]. However, both studies included only a small number of patients, did not distinguish between fasciocutaneous free flaps (FFFs) and perforator free flaps (PFFs), despite their different vascular anatomies, and did not provide distinct flap perfusion parameters, such as flap blood flow and hemoglobin oxygen saturation [13, 16–23].

Even though microvascular free flap reconstruction is routinely performed, little is known about the physiology of flap perfusion [24]. Understanding the influence of blood pressure on flap perfusion may help to improve hemodynamic management during microvascular free flap reconstruction and thus ensure flap perfusion and the monitoring of free flaps [2, 3, 12, 13, 16, 25, 26].

Therefore, this study was intended to investigate the influence of blood pressure on microvascular free flap perfusion.

## Materials and methods

### Study population

The local ethics committee of the Medical Faculty RWTH Aachen University (EK 309 − 20) approved this retrospective study based on data collected for routine clinical purposes.

The study population consisted of 244 patients who underwent head and neck reconstruction with a FFF (radial free forearm flap) or a PFF (anterolateral thigh flap or fibula free flap) after malignant or nonmalignant diseases in our Department of Oral and Maxillofacial Surgery between 2011 and 2020. Patient exclusion criteria were incomplete data and age under 18 years.

The baseline characteristics of the study population were defined as follows: surgery duration was calculated as the time interval between the first incision and the last suture; flap ischemia duration was calculated as the time interval between the cessation of flap perfusion at the donor site and the restoration of flap perfusion at the recipient site; smoking status was defined as actual or past daily smoking for a period of at least six months; prior neck dissection was defined as present if the patient had undergone anatomic dissection of the recipient vessel in terms of neck dissection; irradiation status was defined as present if the patient had undergone irradiation to the recipient vessel area in terms of neck irradiation; flap revision was defined as prevalent if the patient underwent surgical revision of the anastomosis; and flap success was absent if the patient had the flap removed due to flap necrosis [27].

All microvascular reconstructive procedures were conducted under general anesthesia, with arterial anastomoses being created in an end-to-end fashion, and venous anastomoses being created in an end-to-side or end-to-end fashion. Patients remained in the intensive care unit postoperatively until at least the morning following surgery, with invasive mechanical ventilation, analgosedation, invasive arterial blood pressure measurement, and blood pressure regulation via central venous norepinephrine as needed (target systolic blood pressure above 125 mmHg).

Preoperative blood pressure values were determined via non-invasive blood pressure measurement before the induction of anesthesia, taking the mean of all available measurements, and the intra- and postoperative blood pressure values related to flap perfusion measurement were determined via invasive arterial blood pressure measurement. Mean arterial blood pressure (MBP) was defined according to the following commonly used formula: MBP = diastolic blood pressure (DBP) + 1/3 x (systolic blood pressure (SBP)– DBP) [28].

### Flap perfusion measurement data

Flap perfusion measurement data were obtained with the Oxygen-2-see (O2C) analysis system (O2C Oxygen-to-see, LEA Medizintechnik, Giesen, Germany) intraoperatively (after the release of the anastomosis in the operating room) and postoperatively (on the first postoperative morning in the intensive care unit) for 10s, with a lead time of 4s with ambient light compensation control at 8 and 2 mm tissue depths, and with the probe placed centrally on the dried skin portion of the flap in a sterile sheath. The determination of flap blood flow (arbitrary units [AU]) was performed using laser Doppler spectroscopy (830 nm; 30 mW), and the value was calculated by evaluating the Doppler shift of the laser light due to erythrocyte movement, which was calculated as the product of erythrocyte quantity and erythrocyte velocity [13, 29]. Hemoglobin concentration (AU) and oxygen saturation (%) were determined using white light spectroscopy (500–800 nm; 50 W), and the values were calculated by evaluating the sum of the absorbances of the white light and the color change of the white light in comparison to reference hemoglobin spectra with known oxygen saturation levels, respectively [13, 29]. For further analysis, the mean values of the measurements at 8 and 2 mm tissue depths were calculated.

### Statistical analysis

Patients were grouped according to flap type (FFF or PFF). Associations between blood pressure values (i.e., SBP, DBP, and MBP) and flap perfusion parameters were analyzed separately for each flap type by calculating the Spearman correlation coefficient. In the case of significant results, associations were further analyzed using multiple linear regression analysis, adjusting for flap ischemia duration (min), flap size (cm²), preoperative systolic, diastolic, or mean arterial blood pressure value (mmHg), preoperative hypertension (yes vs. no), and administered catecholamine dose (µg/min per kg) in FFFs and adjusting for flap ischemia duration (min), flap size (cm²), flap type (anterolateral thigh flap vs. fibula free flap), preoperative systolic, diastolic, or mean arterial blood pressure value (mmHg), preoperative hypertension (yes vs. no), and administered catecholamine dose (µg/min per kg) in PFFs. Values of *p* < 0.05 were considered statistically significant. The statistical analysis was performed using SPSS Version 28 (SPSS, IBM, New York, USA) and graphics were created using GraphPad Prism 4 (GraphPad Prism, GraphPad Software, Bosten, USA).

## Results

### Clinical characteristics of the study population

The study population consisted of 244 patients (121 patients reconstructed with FFFs and 123 patients reconstructed with PFFs [90 patients reconstructed with an anterolateral thigh flap and 33 patients reconstructed with a fibula free flap]) (Table 1). Flap revision was performed in seven FFFs and four PFFs due to venous congestion and in one PFF due to arterial insufficiency.

**Table 1:** Clinical characteristics of the study population

Variable	All (*n* = 244)	FFF (*n* = 121)	PFF (*n* = 123)
***Sex*** *(n)*			
male	125 (51.2%)	62 (51.2%)	63 (51.2%)
female	119 (48.8%)	59 (48.8%)	60 (48.8%)
***Age*** *(years)*	64.0 (18.0)	65.0 (17.0)	61.0 (20.0)
***BMI*** *(kg/m²)*	24.4 (5.9)	24.9 (6.5)	23.8 (5.4)
***ASA*** *(n)*			
1 + 2	139 (57.0%)	73 (60.3%)	66 (53.7%)
3 + 4	105 (43.0%)	48 (39.7%)	57 (46.3%)
***Flap location*** *(n)*			
tongue	37 (15.2%)	28 (23.1%)	9 (7.3%)
floor of mouth	52 (21.3%)	33 (27.3%)	19 (15.4%)
mandible	63 (25.8%)	17 (14.0%)	46 (37.4%)
maxilla + hard palate	30 (12.3%)	12 (9.9%)	18 (14.6%)
cheek	18 (7.4%)	11 (9.1%)	7 (5.7%)
soft palate	11 (4.5%)	8 (6.6%)	3 (2.4%)
extraoral	33 (13.5%)	12 (9.9%)	21 (17.1%)
***Arterial anastomosis recipient vessel*** *(n)*			
ECA	19 (7.8%)	4 (3.3%)	15 (12.2%)
FAA	95 (38.9%)	45 (37.2%)	50 (40.7%)
LIA	14 (5.7%)	4 (3.3%)	10 (8.1%)
STA	116 (47.5%)	68 (56.2%)	48 (39.0%)
***Surgery duration*** *(min)*	550.0 (170.0)	515.0 (174.0)	574.0 (156.0)
***Flap ischemia duration*** *(min)*	108.0 (35.0)	108.0 (34.0)	108.0 (37.0)
***Diabetes*** *(n)*			
no	205 (84.0%)	101 (83.5%)	104 (84.6%)
yes	39 (16.0%)	20 (16.5%)	19 (15.4%)
***Arterial hypertension*** *(n)*			
no	157 (64.3%)	72 (59.5%)	85 (69.1%)
yes	87 (35.7%)	49 (40.5%)	38 (30.9%)
**Smoking status** *(n)*			
no	151 (61.9%)	74 (61.2%)	77 (62.6%)
yes	93 (38.1%)	47 (38.8%)	46 (37.4%)
**Prior neck dissection** *(n)*			
no	185 (75.8%)	102 (84.3%)	83 (67.5%)
yes	59 (24.2%)	19 (15.7%)	40 (32.5%)
**Prior neck irradiation** *(n)*			
no	212 (86.9%)	114 (94.2%)	98 (79.7%)
yes	32 (13.1%)	7 (5.8%)	25 (20.3%)
**Flap survival** *(n)*			
no	4 (1.6%)	1 (0.8%)	3 (2.4%)
yes	240 (98.4%)	120 (99.2%)	120 (97.6%)
**Flap revision** *(n)*			
no	232 (95.1%)	114 (94.2%)	118 (95.9%)
yes	12 (4.9%)	7 (5.8%)	5 (4.1%)

Parameters are indicated as numbers (with percentage) for categorical data (sex, ASA, flap location, arterial anastomosis recipient vessel, diabetes, arterial hypertension, smoking status, prior neck dissection, prior neck irradiation, flap survival, flap revision) or median (with interquartile range) for metric data (age, BMI, surgery duration, flap ischemia duration) (separately described for all patients, patients reconstructed with a FFF, and patients reconstructed with a PFF); abbreviations: FFF = fasciocutaneous free flap, PFF = perforator free flap, BMI = body mass index, ASA = American Society of Anesthesiologists score, ECA = external carotid artery, FAA = facial artery, LIA = lingual artery, STA = superior thyroid artery

### Blood pressure values

The preoperative median blood pressure values in patients reconstructed with FFFs or PFFs were as follows: the SBP values were both 134.0 mmHg; the DBP values were 74.0 mmHg and 76.0 mmHg; and the MBP values were 95.0 mmHg and 96.0 mmHg, respectively (Table 2). The intraoperative median blood pressure values in relation to intraoperative flap perfusion measurement in patients reconstructed with FFFs or PFFs were as follows: the SBP values were both 125.0 mmHg; the DBP values were both 60.0 mmHg; and the MBP values were 80.3 mmHg and 80.0 mmHg, respectively. The postoperative median blood pressure values related to postoperative flap perfusion measurement in patients reconstructed with FFFs or PFFs were as follows: the SBP values were both 133.0 mmHg; the DBP values were 57.0 mmHg and 59.0 mmHg; and the MBP values were 81.3 mmHg and 83.3 mmHg, respectively.

**Table 2:** Blood pressure values

Variable	FFF (*n* = 121)	PFF (*n* = 123)
**Preoperative values**		
**SBP** (mmHg)	134.0 (30.0)	134.0 (34.0)
**DBP** (mmHg)	74.0 (13.0)	76.0 (16.0)
**MBP** (mmHg)	95.0 (13.0)	96.0 (20.0)
**Intraoperative values**		
**SBP** (mmHg)	125.0 (15.0)	125.0 (22.0)
**DBP** (mmHg)	60.0 (10.0)	60.0 (11.0)
**MBP** (mmHg)	80.3 (13.0)	80.0 (13.0)
**Postoperative values**		
**SBP** (mmHg)	133.0 (21.0)	133.0 (21.0)
**DBP** (mmHg)	57.0 (14.0)	59.0 (13.0)
**MBP** (mmHg)	81.3 (14.0)	83.3 (12.0)

Parameters are indicated as median (with interquartile range) for SBP (mmHg), DBP (mmHg), and MBP (mmHg) according to preoperative values, intraoperative values used as reference values for intraoperative flap perfusion measurement, and postoperative values used as reference values for postoperative flap perfusion measurement (separately described for patients reconstructed with a FFF and patients reconstructed with a PFF); abbreviations: SBP = systolic blood pressure, DBP = diastolic blood pressure, MBP = mean arterial blood pressure, FFF = fasciocutaneous free flap, PFF = perforator free flap

### Association between flap perfusion parameters and blood pressure values in FFFs

Postoperative hemoglobin concentration was negatively correlated with SBP and MBP in FFFs (*r*=-0.272, *p* = 0.003; *r*=-0.199, *p* = 0.029, respectively) (Table 3). Both associations persisted in multivariable testing (*p* = 0.005 and *p* = 0.027, respectively).

**Table 3:** Association between flap perfusion parameters and blood pressure values in FFFs

Variable	SBP (mmHg)		DBP (mmHg)		MBP (mmHg)	
	*r*	*p*-value	*r*	*p*-value	*r*	*p*-value
**Intraoperative measurement**						
**Flow** (AU)	0.021	0.815	-0.071	0.436	-0.037	0.686
**Hemoglobin concentration** (AU)	0.078	0.397	0.076	0.408	0.075	0.416
**Hemoglobin oxygen saturation** (%)	-0.027	0.771	-0.106	0.248	-0.058	0.527
**Postoperative measurement**						
**Flow** (AU)	-0.013	0.888	0.084	0.358	0.046	0.615
**Hemoglobin concentration** (AU)	**-0.272**	0.003*	-0.087	0.344	**-0.199**	0.029*
**Hemoglobin oxygen saturation** (%)	-0.052	0.569	0.018	0.847	-0.004	0.963

Parameters are indicated as Spearman correlation coefficient (r) with p-value for patients reconstructed with a FFF; significant p-values are bold (*p < 0.05 upon adjustment for flap ischemia duration (min), flap size (cm²), preoperative blood pressure SBP, DBP, or MBP (mmHg), preoperative arterial hypertension, and administered catecholamine dose (µg/min per kg) in multiple linear regression analysis); abbreviations: SBP = systolic blood pressure, DBP = diastolic blood pressure, MBP = mean arterial blood pressure, FFF = fasciocutaneous free flap

### Association between flap perfusion parameters and blood pressure values in PFFs

**Fig. 1:**
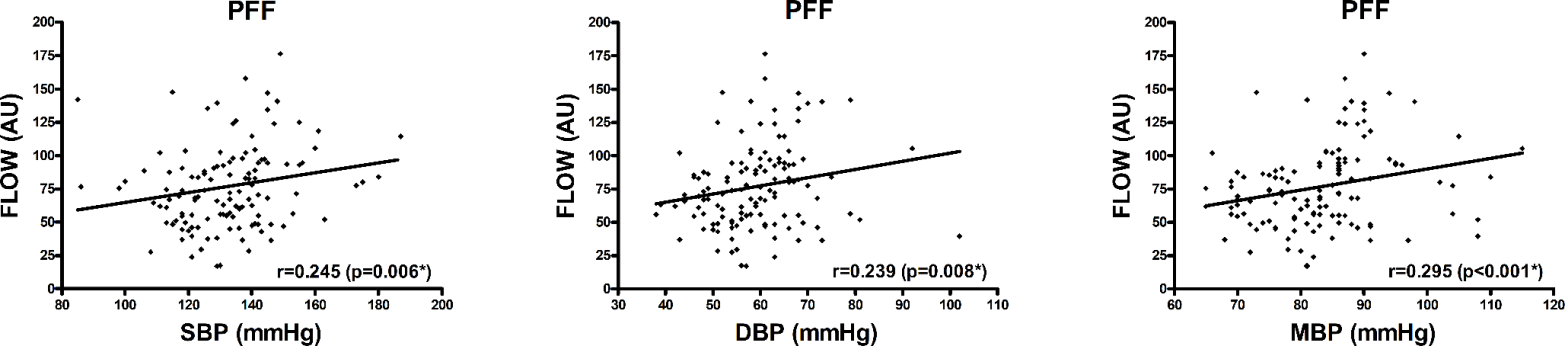
Postoperative blood flow and blood pressure in PFFs Scatter plot for postoperative flap blood flow (AU) and blood pressure (mmHg) in PFFs (separately described for SBP, DBP, and MBP); r and p-value corresponding to Spearman correlation coefficient (*p < 0.05 upon adjustment for flap ischemia duration (min), flap size (cm²), flap type (anterolateral thigh flap vs. fibula free flap), preoperative systolic/diastolic/mean arterial blood pressure (mmHg), preoperative arterial hypertension, and administered catecholamine dose (µg/min per kg) in multiple linear regression analysis); abbreviations: PFF = perforator free flap, AU = arbitrary units

Postoperative flap blood flow was positively correlated with SBP, DBP, and MBP in PFFs (*r* = 0.245, *p* = 0.006; *r* = 0.239, *p* = 0.008; *r* = 0.295, *p* < 0.001, respectively) (Table 4; Fig. 1). All these associations persisted in multivariable testing (*p* = 0.024; *p* = 0.004; *p* = 0.027, respectively). Postoperative hemoglobin concentration was positively correlated with DBP and MBP in PFFs (*r* = 0.298, *p* < 0.001; *r* = 0.212, *p* = 0.019, respectively) (Table 4). Only the association with DBP persisted in multivariable testing (*p* = 0.027). Postoperative hemoglobin oxygen saturation was positively correlated with SBP and MBP in PFFs (*r* = 0.253, *p* = 0.005; *r* = 0.198, *p* = 0.036, respectively). Only the association with SBP persisted in multivariable testing (*p* = 0.005).

**Table 4:** Association between flap perfusion parameters and blood pressure values in PFFs

Variable	SBP (mmHg)		DBP (mmHg)		MBP (mmHg)	
	*r*	*p*-value	*r*	*p*-value	*r*	*p*-value
**Intraoperative measurement**						
**Flow** (AU)	-0.023	0.804	0.073	0.412	0.043	0.640
**Hemoglobin concentration** (AU)	0.056	0.539	0.052	0.565	0.061	0.502
**Hemoglobin oxygen saturation** (%)	-0.107	0.237	-0.016	0.860	-0.079	0.386
**Postoperative measurement**						
**Flow** (AU)	**0.245**	0.006*	**0.239**	0.008*	**0.295**	< 0.001*
**Hemoglobin concentration** (AU)	0.053	0.564	**0.298**	< 0.001*	**0.212**	**0.019**
**Hemoglobin oxygen saturation** (%)	**0.253**	0.005*	0.068	0.454	**0.189**	**0.036**

Parameters are indicated as Spearman correlation coefficient (r) with p-value for patients reconstructed with a PFF; significant p-values are bold (*p < 0.05 upon adjustment for flap ischemia duration (min), flap size (cm²), flap type (anterolateral thigh flap vs. fibula free flap), preoperative blood pressure SBP, DBP, or MBP (mmHg), preoperative arterial hypertension, and administered catecholamine dose (µg/min per kg) in multiple linear regression analysis); abbreviations: SBP = systolic blood pressure, DBP = diastolic blood pressure, MBP = mean arterial blood pressure, PFF = perforator free flap

## Discussion

This study investigated the influence of blood pressure on microvascular free flap perfusion, asknowledge about the influence of blood pressure on flap perfusion could impact both hemodynamic management, to balance the requirements of the general body circulation and the flap tissue perfusion, and flap monitoring, to detect vascular flap compromise by measuring flap perfusion with the O2C analysis system [2, 3, 12, 13, 15, 16, 25, 26, 30].

In this study, microvascular free flaps were investigated separately for FFFs and PFFs, as these flap types differ in their vascular anatomies and the flap perfusion parameters measured with the O2C analysis system [13, 16, 19–22]. In addition, blood pressure was examined separately in terms of SBP, DBP, and MBP, as these may all separately influence flap perfusion and, thus, be of interest regarding intraoperative and postoperative hemodynamic management [28, 31–33].

This study demonstrated that flap perfusion in PFFs was influenced by blood pressure, as postoperative flap blood flow was positively associated with SBP, DBP, and MBP.

These findings stand in contrast with those of a study that also investigated the influence of blood pressure on microvascular free flap perfusion, but that study included only a small number of patients and the measurement methods used were not comparable [18]. The findings of this study were expected given a theoretical model indicating that the blood pressure gradient in the microcirculatory vasculature of the flap is a major determinant of tissue perfusion and flap perfusion, along with vessel radius, blood viscosity, and vessel length, with the effect being exaggerated in microvascular free flaps due to their denervation and loss of neural adrenergic vascular tone control after flap harvesting [2, 4, 6, 11]. However, blood pressure, when measured as invasive arterial blood pressure, is not equivalent to the blood pressure gradient in the microcirculatory vasculature of the flap; still it likely reflects this blood pressure gradient, as the blood pressure in the venous vessel downstream of the microcirculatory vasculature of the flap is relatively low and, thus, negligible [1, 6, 34, 35]. The fact that these associations were only observed in PFFs and not in FFFs may be due to the different vascular anatomies of the two flap types, with PFFs (i.e. anterolateral thigh flaps and fibula free flaps) being more susceptible to blood pressure due to serial flow resistances based on their fewer perforator vessels, with a decreasing total vessel diameter from the flap pedicle to the microcirculation; in contrast to FFFs, which are less susceptible to blood pressure influences due to parallel flow resistances based on their multiple perforator vessels, with an increasing vessel diameter from the flap pedicle to the microcirculation [19–23]. In general, the associations were weak in quantitative terms but persisted in multivariable analysis after adjustment for potentially confounding variables, implying in clinical terms that blood pressure is only one of several factors influencing flap perfusion [36]. In contrast, the positive association between postoperative hemoglobin oxygen saturation and MBP in PFFs found in this study, which is consistent with the results of a previous study showing a positive correlation between tissue partial oxygen pressure in free flaps and MBP without adjustment for confounding factors, did not persist in multivariable testing [17]. The positive association between hemoglobin oxygen saturation and blood pressure, specifically SBP, in PPFs may be due to the general dependence of hemoglobin oxygen saturation on flap blood flow [16].

Unexpectedly, the association between postoperative hemoglobin concentration and blood pressure was negative in FFFs with respect to SBP and MBP and positive in PFFs with respect to DBP. Regarding flap viability and survival, as well as flap monitoring based on the measurement of flap perfusion with the O2C analysis system in relation to predefined threshold values (i.e., absolute threshold values for flap blood flow and hemoglobin oxygen saturation and relative threshold values for hemoglobin concentration), however, the perfusion parameter hemoglobin concentration plays a subordinate role [6, 11, 13, 15, 16].

In general, the observation that only postoperative flap perfusion was affected by blood pressure may be attributable to an adaption period for the flap tissue after reperfusion with rearrangement of ischemia-induced flap tissue changes [6, 37].

This study has several limitations, as flap perfusion was only measured at two timepoints, which represent only a short time period after flap reperfusion [38]. However, data on independent variables, such as blood pressure values, and presumably confounding variables, such as catecholamine dose administered, were only available for the two timepoints used in this study [31]. In addition, measuring flap perfusion at only one spot in the center of the flap, which represents only a small portion of the flap microcirculation, is a limitation, as flap microcirculation may vary throughout the flap tissue due to vascular shunts and local metabolic changes [14, 39]. However, measuring flap perfusion with the O2C analysis system at a central spot of the flap is common in microvascular free flap monitoring [13, 16]. In addition, several potentially confounding factors regarding flap perfusion, such as the vascular anatomy of the cervical recipient vessels in terms of vessel length and diameter or pretreatment of the cervical recipient vessels in terms of prior neck dissection or neck irradiation, cannot be excluded. In general, the blood pressure values were not extremely low or high, which may limit the applicability of the study results. It should also be mentioned that the accuracy of the formula used to calculate the mean blood pressure is influenced by the heart rate of the patients, which was not taken into account and could therefore confound the results of this study.

This study sheds light on microvascular flap perfusion and shows that flap perfusion is partially dependent on blood pressure, notably flap blood flow in PFFs. In terms of clinical implications, this emphasizes that blood pressure could be a parameter used in controlling free flap perfusion to ensure flap survival by increasing blood pressure, as decreased flap perfusion has been linked to flap failure [2, 6, 8, 11, 13–16]. In addition, the choice of PFFs for microvascular head and neck reconstruction in patients who are expected to have difficult hemodynamic management to ensure stable blood pressure (e.g., patients with cardiovascular comorbidities) may need to be critically evaluated [3, 11, 40]. In addition, blood pressure should be considered as a confounding variable in the context of flap monitoring with the O2C analysis system in terms of flap blood flow and hemoglobin oxygen saturation [13, 16]. Further studies are needed to confirm these results.

## Conclusion

The results of this study indicate that microvascular free flap perfusion in PFFs is partially influenced by blood pressure, as postoperative flap blood flow in PFFs was weakly positively associated with blood pressure in terms of SBP, DBP, and MBP. This emphasizes that blood pressure may serve as an extra control variable for flap perfusion in PFFs, which could help ensure flap viability and survival. It also implies that blood pressure should be considered as a potential confounding variable in the context of flap monitoring with the O2C analysis system with regard to absolute threshold values indicating vascular flap compromise, specifically flap blood flow and hemoglobin oxygen saturation.

## Data Availability

The datasets used and/or analyzed during the current study are under further analysis and are available from the corresponding author on reasonable request.

## References

[1] Pattani KM, Byrne P, Boahene K, Richmon J (2010) What makes a good flap go bad? A critical analysis of the literature of intraoperative factors related to free flap failure. Laryngoscope 120:717–723. 10.1002/lary.20825 20205243 10.1002/lary.20825

[2] Wax MK, Azzi J (2018) Perioperative considerations in free flap surgery: A review of pressors and anticoagulation. Oral Oncol 83:154–157. 10.1016/j.oraloncology.2018.06.025 30098772 10.1016/j.oraloncology.2018.06.025

[3] Abouyared M, Katz AP, Ein L, Ketner J, Sargi Z, Nicolli E, Leibowitz JM (2019) Controversies in free tissue transfer for head and neck cancer: A review of the literature. Head Neck 41:3457–3463. 10.1002/hed.25853 31286627 10.1002/hed.25853

[4] Goh CSL, Ng MJM, Song DH, Ooi ASH (2019) Perioperative vasopressor use in free flap surgery: A systematic review and Meta-Analysis. J Reconstr Microsurg 35:529–540. 10.1055/s-0039-1687914 31042803 10.1055/s-0039-1687914

[5] Wang K-Y, Lin Y-S, Chen L-W, Yang K-C, Huang W-C, Liu W-C (2020) Risk of free flap failure in head and neck reconstruction: analysis of 21,548 cases from A nationwide database. Ann Plast Surg 84:S3–S6. 10.1097/SAP.0000000000002180 31833882 10.1097/SAP.0000000000002180

[6] Quinlan J (2006) Anaesthesia for reconstructive surgery. Anaesth Intensive Care Med 7:31–35. 10.1383/anes.2006.7.1.31

[7] Hand WR, McSwain JR, McEvoy MD, Wolf B, Algendy AA, Parks MD et al (2015) Characteristics and intraoperative treatments associated with head and neck free tissue transfer complications and failures. Otolaryngol Head Neck Surg 152:480–487. 10.1177/0194599814564366 25550221 10.1177/0194599814564366 PMC4516157

[8] Kass JL, Lakha S, Levin MA, Joseph T, Lin H-M, Genden EM et al (2018) Intraoperative hypotension and flap loss in free tissue transfer surgery of the head and neck. Head Neck 40:2334–2339. 10.1002/hed.25190 30230116 10.1002/hed.25190

[9] Naik AN, Freeman T, Li MM, Marshall S, Tamaki A, Ozer E et al (2020) The use of vasopressor agents in free tissue transfer for head and neck reconstruction: current trends and review of the literature. Front Pharmacol 11:1248. 10.3389/fphar.2020.01248 32982724 10.3389/fphar.2020.01248 PMC7485519

[10] Zhang L, Yu Y, Xue J, Lei W, Huang Y, Li Y, Sun J (2021) Effect of deliberate hypotension on regional cerebral oxygen saturation during functional endoscopic sinus surgery: A randomized controlled trial. Front Surg 8:681471. 10.3389/fsurg.2021.681471 34568412 10.3389/fsurg.2021.681471 PMC8456080

[11] McCauley P, Moore M, Duggan E (2022) Anaesthesia for reconstructive free flap surgery for head and neck cancer. Br J Hosp Med (Lond) 83:1–9. 10.12968/hmed.2021.0668 35653311 10.12968/hmed.2021.0668

[12] Burkhard J-P, Wepfer A, Löffel LM, Bachmann KF, Wuethrich PY (2023) The role of intraoperative and early postoperative blood pressure variations, fluid balance and inotropics in fibula free flap head and neck reconstruction: A retrospective analysis. J Clin Med 12. 10.3390/jcm12247753 10.3390/jcm12247753 PMC10743382 38137822

[13] Hölzle F, Loeffelbein DJ, Nolte D, Wolff K-D (2006) Free flap monitoring using simultaneous non-invasive laser doppler flowmetry and tissue spectrophotometry. J Craniomaxillofac Surg 34:25–33. 10.1016/j.jcms.2005.07.010 16343915 10.1016/j.jcms.2005.07.010

[14] Miyamoto S, Minabe T, Harii K (2008) Effect of recipient arterial blood inflow on free flap survival area. Plast Reconstr Surg 121:505–513. 10.1097/01.prs.0000299185.32881.55 18300969 10.1097/01.prs.0000299185.32881.55

[15] Abdel-Galil K, Mitchell D (2009) Postoperative monitoring of microsurgical free tissue transfers for head and neck reconstruction: a systematic review of current techniques—part I. Non-invasive techniques. Br J Oral Maxillofac Surg 47:351–355. 10.1016/j.bjoms.2008.11.013 19144453 10.1016/j.bjoms.2008.11.013

[16] Hölzle F, Rau A, Loeffelbein DJ, Mücke T, Kesting MR, Wolff K-D (2010) Results of monitoring fasciocutaneous, myocutaneous, osteocutaneous and perforator flaps: 4-year experience with 166 cases. Int J Oral Maxillofac Surg 39:21–28. 10.1016/j.ijom.2009.10.012 19944567 10.1016/j.ijom.2009.10.012

[17] Schrey A, Kinnunen I, Vahlberg T, Minn H, Grenman R, Taittonen M, Aitasalo K (2011) Blood pressure and free flap oxygenation in head and neck cancer patients. Acta Otolaryngol 131:757–763. 10.3109/00016489.2011.554438 21413842 10.3109/00016489.2011.554438

[18] Massaro A, Gomez J, Weyh AM, Bunnell A, Warrick M, Pirgousis P, Fernandes R (2021) Serial perioperative assessment of free flap perfusion with laser angiography. Craniomaxillofac Trauma Reconstr 14:16–22. 10.1177/1943387520930608 33613831 10.1177/1943387520930608 PMC7868510

[19] Coscia V, Rubino C (2005) Hemodynamic enhancement in reconstructive surgery: mathematical model and clinical findings. Math Comput Model 42:1151–1161. 10.1016/j.mcm.2004.11.005

[20] Rubino C, Coscia V, Cavazzuti AM, Canu V (2006) Haemodynamic enhancement in perforator flaps: the inversion phenomenon and its clinical significance. A study of the relation of blood velocity and flow between pedicle and perforator vessels in perforator flaps. J Plast Reconstr Aesthet Surg 59:636–643. 10.1016/j.bjps.2005.07.010 16817260 10.1016/j.bjps.2005.07.010

[21] Yu P (2014) Inverse relationship of the anterolateral and anteromedial thigh flap perforator anatomy. J Reconstr Microsurg 30:463–468. 10.1055/s-0034-1370361 24995393 10.1055/s-0034-1370361

[22] Sham E, Masia JA, Reddy TJ (2018) Vascular analysis of radial artery perforator flaps. Ann Maxillofac Surg 8:66–72. 10.4103/ams.ams_1_18 29963427 10.4103/ams.ams_1_18 PMC6018282

[23] Weyh AM, Fernandes RP (2021) Narrative review: fibula free flap, indications, tips, and pitfalls. Front Oral Maxillofac Med 3:4. 10.21037/fomm-20-43

[24] Lorenzetti F, Suominen S, Tukiainen E, Kuokkanen H, Suominen E, Vuola J, Asko-Seljavaara S (2001) Evaluation of blood flow in free microvascular flaps. J Reconstr Microsurg 17:163–167. 10.1055/s-2001-14347 11336147 10.1055/s-2001-14347

[25] Hagau N, Longrois D (2009) Anesthesia for free vascularized tissue transfer. Microsurgery 29:161–167. 10.1002/micr.20584 18946883 10.1002/micr.20584

[26] Mastrolonardo EV, Lu JS, Elliott Z, Knops A, Philips R, Urdang Z et al (2023) Evaluating the impact of hemodynamic support measures on head and neck free tissue transfer outcomes: A population-based analysis. Oral Oncol 143:106461. 10.1016/j.oraloncology.2023.106461 37331035 10.1016/j.oraloncology.2023.106461

[27] Latza U, Hoffmann W, Terschüren C, Chang-Claude J, Kreuzer M, Schaffrath Rosario A et al (2005) Rauchen Als möglicher confounder in epidemiologischen studien: standardisierung der Erhebung, quantifizierung und analyse (Smoking as potential confounder in German epidemiological studies: standardization of assessment, quantification, and analysis). Gesundheitswesen 67:795–802. 10.1055/s-2005-858807 16308812 10.1055/s-2005-858807

[28] Whelton PK, Carey RM, Aronow WS, Casey DE, Collins KJ, Himmelfarb CD, AAPA/ABC/ACPM/AGS/APhA/ASH et al (2018) /ASPC/NMA/PCNA Guideline for the prevention, detection, evaluation, and management of high blood pressure in adults: A report of the American college of cardiology/american heart association task force on clinical practice guidelines. Hypertension 71:e127–e248. 10.1016/j.jacc.2017.11.006 10.1016/j.jacc.2017.11.006 29146535

[29] Beckert S, Witte MB, Königsrainer A, Coerper S (2004) The impact of the Micro-Lightguide O2C for the quantification of tissue ischemia in diabetic foot ulcers. Diabetes Care 27:2863–2867. 10.2337/diacare.27.12.2863 15562198 10.2337/diacare.27.12.2863

[30] Sweeny L, Curry J, Crawley M, Cave T, Stewart M, Luginbuhl A et al (2020) Factors impacting successful salvage of the failing free flap. Head Neck. 10.1002/hed.26427 32844522 10.1002/hed.26427

[31] de Backer D, Foulon P (2019) Minimizing catecholamines and optimizing perfusion. Crit Care 23:149. 10.1186/s13054-019-2433-6 31200777 10.1186/s13054-019-2433-6 PMC6570631

[32] Sun J, Yuan J, Li B (2021) SBP is superior to MAP to reflect tissue perfusion and hemodynamic abnormality perioperatively. Front Physiol 12:705558. 10.3389/fphys.2021.705558 34594235 10.3389/fphys.2021.705558 PMC8476970

[33] Saugel B, Sessler DI (2021) Perioperative blood pressure management. Anesthesiology 134:250–261. 10.1097/ALN.0000000000003610 33206118 10.1097/ALN.0000000000003610

[34] Patel SA, Keller A (2008) A theoretical model describing arterial flow in the DIEP flap related to number and size of perforator vessels. J Plast Reconstr Aesthet Surg 61:1316–1320 discussion 1320. 10.1016/j.bjps.2007.08.020 18243080 10.1016/j.bjps.2007.08.020

[35] Nakamura Y, Takanari K, Nakamura R, Ono M, Uchibori T, Hishida M et al (2020) Correlation between blood flow, tissue volume and microvessel density in the flap. Nagoya J Med Sci 82:291–300. 10.18999/nagjms.82.2.291 32581408 10.18999/nagjms.82.2.291 PMC7276411

[36] Schober P, Boer C, Schwarte LA (2018) Correlation coefficients: appropriate use and interpretation. Anesth Analg 126:1763–1768. 10.1213/ANE.0000000000002864 29481436 10.1213/ANE.0000000000002864

[37] Carroll WR, Esclamado RM (2000) Ischemia/reperfusion injury in microvascular surgery. Head Neck 22:700–713. 10.1002/1097-0347(200010)22:7<;700:AID-HED10>;3.0.CO;2-H 11002326 10.1002/1097-0347(200010)22:7<700::aid-hed10>3.0.co;2-h

[38] Eley KA, Young JD, Watt-Smith SR (2012) Epinephrine, norepinephrine, Dobutamine, and dopexamine effects on free flap skin blood flow. Plast Reconstr Surg 130:564–570. 10.1097/PRS.0b013e31825dbf73 22929242 10.1097/PRS.0b013e31825dbf73

[39] Dusseldorp JR, Pennington DG (2014) Quantifying blood flow in the DIEP flap: an ultrasonographic study. Plast Reconstr Surg Glob Open 2:e228. 10.1097/GOX.0000000000000191 25426345 10.1097/GOX.0000000000000191 PMC4236373

[40] Brinkman JN, Derks LH, Klimek M, Mureau MAM (2013) Perioperative fluid management and use of vasoactive and antithrombotic agents in free flap surgery: a literature review and clinical recommendations. J Reconstr Microsurg 29:357–366. 10.1055/s-0033-1343955 23599215 10.1055/s-0033-1343955

